# Supplementary material for: Aggregation, Cytotoxicity and DNA Binding in a Series of Calix[4]arene Amphiphile Containing Aminotriazole Groups
Source: Pharmaceuticals (Basel). 2023 May 5;16(5):699. doi: 10.3390/ph16050699 (PMC10224245; doi:10.3390/ph16050699)
Supplement: Supplementary file 1 [file pharmaceuticals-16-00699-s001.zip › pharmaceuticals-2338621-supplementary.pdf]

Supplementary Materials

Aggregation, cytotoxicity and DNA binding in a series of ca-lix[4]arene amphiphiles containing aminotriazole groups

Diana Mironova[1,*], Egor Makarov[1], Islamiya Bilukova[1], Kevser Akyol[1], Elsa Sultanova[1], Vladimir Evtugyn[2], Damir Davletshin[3], Elvina Gilyazova[3], Emil Bulatov[3], Vladimir Burilov[1], Svetlana Solovieva[4] and Igor Antipin[1]

[1] Alexander Butlerov Institute of Chemistry, Kazan Federal University, 18 Kremlevskaya Str., 420008 Kazan, Russia
[2] Interdisciplinary Center for Analytical Microscopy, Kazan Federal University, 18 Kremlevskaya Str., 420008 Kazan, Russia
[3] Institute of Fundamental Medicine and Biology, Kazan Federal University, 18 Kremlevskaya Str., 420008 Kazan, Russia
[4] Arbuzov Institute of Organic and Physical Chemistry, FRC Kazan Scientific Center of RAS, 8 Arbuzov Str., 420088 Kazan, Russia

* Correspondence: mir_din@mail.ru

Characterization Methods

^{1}H and ^{13}C NMR spectra as well as 2D ^{1}H-^{1}H NOESY were recorded on Bruker Avance 400 Nanobay (Bruker Corporation, Billerica, MA, USA) with signals from residual protons of $CDCl_3$, D_2O or DMSO-d6 as internal standard.

The melting points were measured using the Optimelt MPA100 melting point apparatus (Stanford Research Systems, Sunnyvale, CA, USA).

IR spectra in KBr pellets were recorded on a Bruker Vector-22 spectrometer (Bruker Corporation, Billerica, MA, USA).

High-resolution mass spectra with electrospray ionization (HRESIMS) were obtained on an Agilent iFunnel 6550 Q-TOF LC/MS (Agilent Technologies, Santa Clara, CA, USA) in positive or negative mode. Carrier gas-nitrogen, temperature 300 ˚C, carrier flow rate 12 l × min^{-1}, nebulizer pressure 275 kPa, funnel voltage 3500 V, capillary voltage 500 V, total ion current recording mode, 100–3000 m/z mass range, scanning speed 7 spectra × s^{-1}.

Syntheses procedure

Synthesis of 5,11,17,23-tetra(4-phtalimidomethyl-1,2,3-triazol-1-yl)-25,26,27,28-tetratetradecyloxycalix[4]arene (5):

1 mmol of compound 1, 0.76 g (4 mmol) N-propargylphtalimide, 0.012 g (0.06 mmol) CuI and 1.0 g of triethylamine (10 mmol) were dissolved in 30 ml of dry toluene and N2 was bubbled through the solution for 10 minutes and the reaction mixture was stirred at 40 °C for 24 h. The solvent was evaporated in vacuo and the obtained residue was dissolved in CHCl3 (80 ml) and washed with 0.1M disodium EDTA (100 ml) solution and water (2×150 ml). The organic layer was dried over Na2SO4 and the solvent was evaporated in vacuo. The crude product was precipitated in CHCl3/methanol to give product 5 as white powder. Yield 1.50 g (70%); mp 267°C (decomp.). 1H NMR (400 MHz, CDCl3,, 25 °C) δ 7.80 (dd, J=3.1,5.4, 8H, PhtH), 7.75 (s, 4H, TrzH), 7.68 (dd, J=3.1,5.4, 8H, PhtH), 7.01 (s, 8H, ArH), 5.01 (brs, 8H, Trz-CH2-Pht), 4.53 (d, J=13.5, 4H, Ar-CH2-Ar), 3.94 (t, J=7.2, 8H, O-CH2-Ar), 3.28 (d, J=13.5, 4H, Ar-CH2-Ar), 1.91 (m, 16H, -CH2-), 1.66 (brs, 4H, -CH2-), 1.38 (brs, 16H, -CH2-), 1.25 (brs, 60H, -CH2-), 0.87 (t, J=6.7, 12H, -CH3). 13C NMR (101 MHz, CDCl3, 25 °C) δ 167.80, 156.84, 143.05, 135.94, 134.03, 132.22, 131.89, 123.49, 121.60, 120.86, 75.98, 33.09, 32.05, 31.30, 30.31, 30.09, 30.05, 29.94, 29.84, 29.52, 26.39, 22.81, 14.23. FTIR (KBr) ν max cm -1: 2921 (CH), 2851 (CH), 1717 (C=O), 1396 (C-N), 1230 (CH3). HRESI MS (m/z) [M+H]+, [M+2H]2+: calcd. for [C128H161N16O12]+: 2115.2508, found: 2115.2503; calcd. for [C128H162N16O12]2+: 1058.1290, found: 1058.1295.

Synthesis of 5,11,17,23-tetra(4-phtalimidomethyl-1,2,3-triazol-1-yl)-25,26,27,28-tetrahydroxycalix[4]arene (6):

1 mmol of compound 2, 0.76 g (4 mmol) N-propargylphtalimide, 0.012 g (0.06 mmol) CuI and 3.0 g of triethylamine (30 mmol) were dissolved in 30 ml of dry toluene/DMF solution (5:1) and N2 was bubbled through the mixture for 10 minutes and then reaction mixture was stirred at 40 °C for 24 h. The solvent was evaporated in vacuo. and obtained residue was treated with 1M hydrochloric acid solution and filtered. The precipitate was successively washed with 0.1M disodium EDTA solution (100 ml), H2O (2x150) and methanol (75 ml) to give product 6 as brown powder. Yield 1.21 g (89%); mp 218 °C (decomp.). 1H NMR (400 MHz, DMSO-d6, 25 °C)

δ 8.39 (s, 4H, Trz-H), 7.87 (m, Pht, 16H) 7.61 (s, 8H, ArH), 4.84 (s, 8H, Trz-CH2-Pht), 3.55 (brs, 8H, Ar-CH2-Ar). 13C NMR (101 MHz, DMSO-d6, 25 °C) δ 167.34, 153.36, 142.72, 134.52, 131.66, 129.99, 128.61, 123.23, 121.53, 120.94, 32.89, 30.71. FTIR (KBr) ν max cm -1: 3245 (Ar+OH), 1712 (C=O), 1396 (C-N). HRESI MS (m/z) [M+H]: calcd. for [C72H49N16O12]+: 1329.3710, found: 1329.3704.

Synthesis of 5,11,17,23-tetra(4-aminomethyl-1,2,3-triazol-1-yl)- 25,26,27,28-tetratetradecyloxycalix[4]arene tetrahydrochloride (7):

1.0 mmol of compound 5 was dissolved in 100 ml of methanol, and then 19 ml of 64% hydrazine hydrate was added dropwise. The reaction mixture was stirred at room temperature for 24 h. Methanol and hydrazine were evaporated in vacuo and the crude product was treated with 1M hydrochloric acid solution (100 ml) and filtered. The obtained residue was then washed with 75 ml of acetone to give product 7 as white powder. Yield 1.1g (72%); mp. 207 °C.1H NMR (400 MHz, CDCl3,, 25 °C) 7.71 (brs, 4H, TrzH), 7.07 (brs, 8H, ArH), 4.56 (brs, 4H, Ar-CH2-Ar), 3.97 (brs, 16H, Trz-CH2-Pht, O-CH2-), 3.30 (t, J=7.2, 8H, Ar-CH2-Ar), 1.91 (brs, 16H, -CH2-), 1.39 (brs, 32H, -CH2-), 1.26 (brs, 48H, -CH2-), 0.87 (brs, 12H, -CH3). 13C NMR (101 MHz, CDCl3, 25 °C) δ 131.97, 120.63, 77.16, 76.04, 32.09, 30.39, 30.15, 29.98, 29.88, 29.56, 26.46, 22.84, 14.25. FTIR (KBr) ν max cm -1: 2923 (CH), 2852 (CH), 1597 (NH), 1488 (CH), 1468 (CH), 1212 (CH3). HRESI MS (m/z) [M+H]+, [M+2H]2+, [M+3H]3+: calcd. for [C96H153N16O4]+: 1595.2289, found: 1595.2221; calcd. for [C96H154N16O4]2+: 798.1181, found: 798,1186; calcd. for [C96H154N16O4]3+: 532.4151, found: 532,4148.

Synthesis of 5,11,17,23-tetra(4-aminomethyl-1,2,3-triazol-1-yl)- 25,26,27,28-tetrahydroxycalix[4]arene tetrahydrochloride (8):

1.0 mmol of compound 6 was dissolved in 100 ml of methanol, and then 10 ml of 64% hydrazine hydrate was added dropwise. The reaction mixture was stirred at room temperature for 24 h. Methanol and hydrazine were evaporated in vacuo and crude product was precipitated by 40 ml of acetone. Then precipitate was treated by 1 ml of conc. HCl and dissolved in 20 ml of water. After evaporation a crude product was crystallized by 20 ml of methanol to give 8 as beige powder. Yield 0.74 (90%), mp 267 °C (decomp.).

1H NMR (400 MHz, D2O, 25 °C) δ 8.27 (s, 4H, TrzH), 7.49 (s, 8H, ArH), 4.60 (brs, 8H, Ar-CH2-Ar), 4.21 (s, 8H, Trz-CH2-N). 1H NMR (400 MHz, DMSO-d6, 25 °C) δ 8.35 (s, 4H, Trz-H), 7.58 (s, 8H, ArH), 4.40 (d, J=11.8, 4H, Ar-CH2-Ar), 3.94 (s, 8H, Trz-CH2-N), 3.53 (d, J=11.8, 4H, Ar-CH2-Ar). 13C NMR (101 MHz, D2O, 25 °C) δ 148.64, 140.20, 130.07, 128.88, 123.17, 120.41, 33.70, 30.48. FTIR (KBr) ν max cm -1: 2924 (OH+Ar+NH), 1604 (NH), 1492 (CH2), 1457 (CH2). HRESI MS (m/z) [M+H]+, [M+2H]2+: calcd. for [C40H41N16O4]+: 809.3491, found: 809.3482; calcd. for [C40H42N16O4]2+: 405.1782, found: 405.1775.

a)

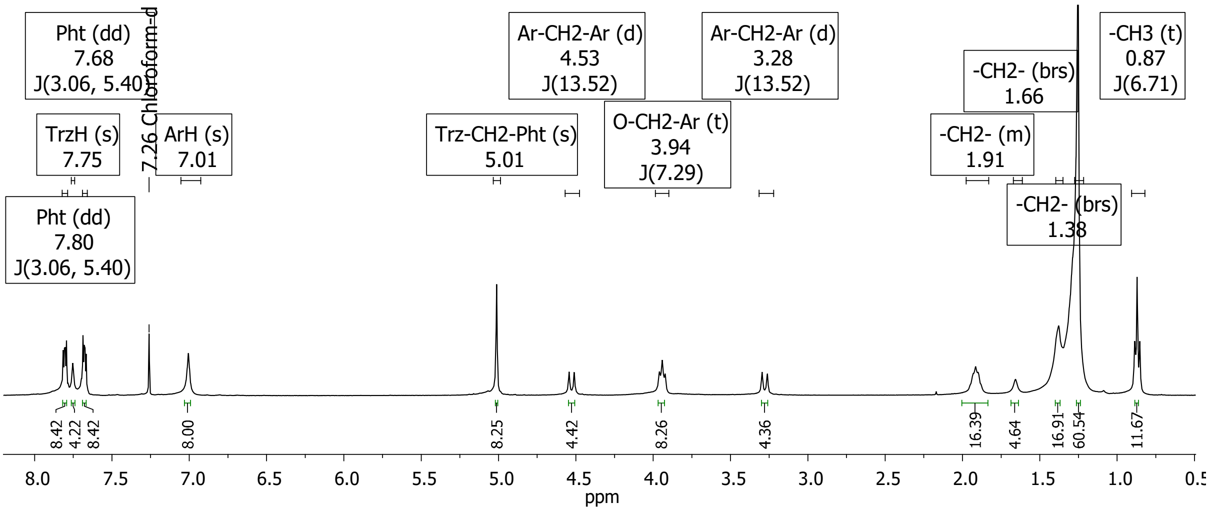
Pht (dd)
7.68
J(3.06, 5.40)
7.26 Chloroform-d
TrzH (s)
7.75
ArH (s)
7.01
Pht (dd)
7.80
J(3.06, 5.40)
Trz-CH2-Pht (s)
5.01
Ar-CH2-Ar (d)
4.53
J(13.52)
O-CH2-Ar (t)
3.94
J(7.29)
Ar-CH2-Ar (d)
3.28
J(13.52)
-CH2- (brs)
1.66
-CH2- (m)
1.91
-CH2- (brs)
1.38
-CH3 (t)
0.87
J(6.71)
8.42
4.22
8.42
8.00
8.25
4.42
8.26
4.36
16.39
4.64
16.91
60.54
11.67
8.0
7.5
7.0
6.5
6.0
5.5
5.0
4.5
4.0
3.5
3.0
2.5
2.0
1.5
1.0
ppm

b)

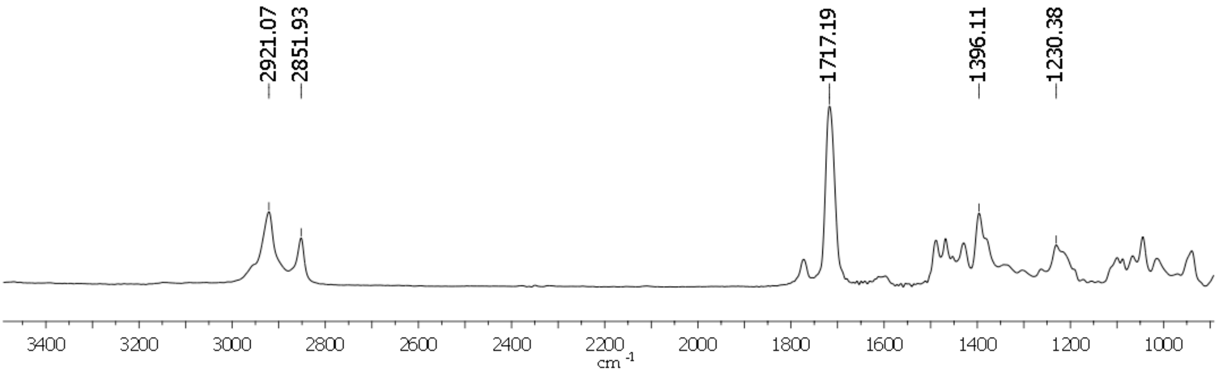
2921.07
2851.93
1717.19
1396.11
1230.38
3400
3200
3000
2800
2600
2400
2200
2000
1800
1600
1400
1200
1000
cm -1

c)

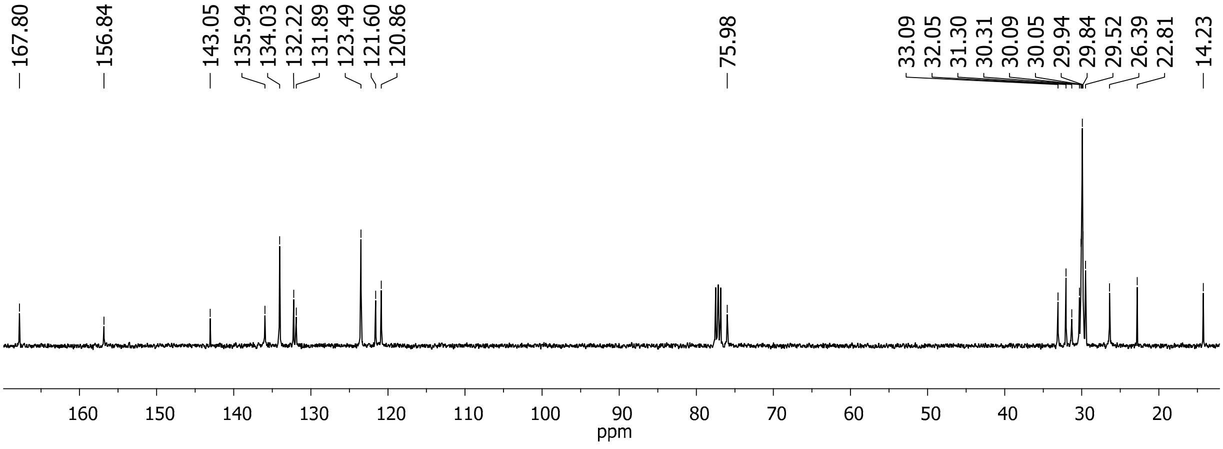
167.80
156.84
143.05
135.94
134.03
132.22
131.89
123.49
121.60
120.86
75.98
33.09
32.05
31.30
30.31
30.09
30.05
29.94
29.84
29.52
26.39
22.81
14.23
160
150
140
130
120
110
100
90
80
70
60
50
40
30
20
ppm

(d)

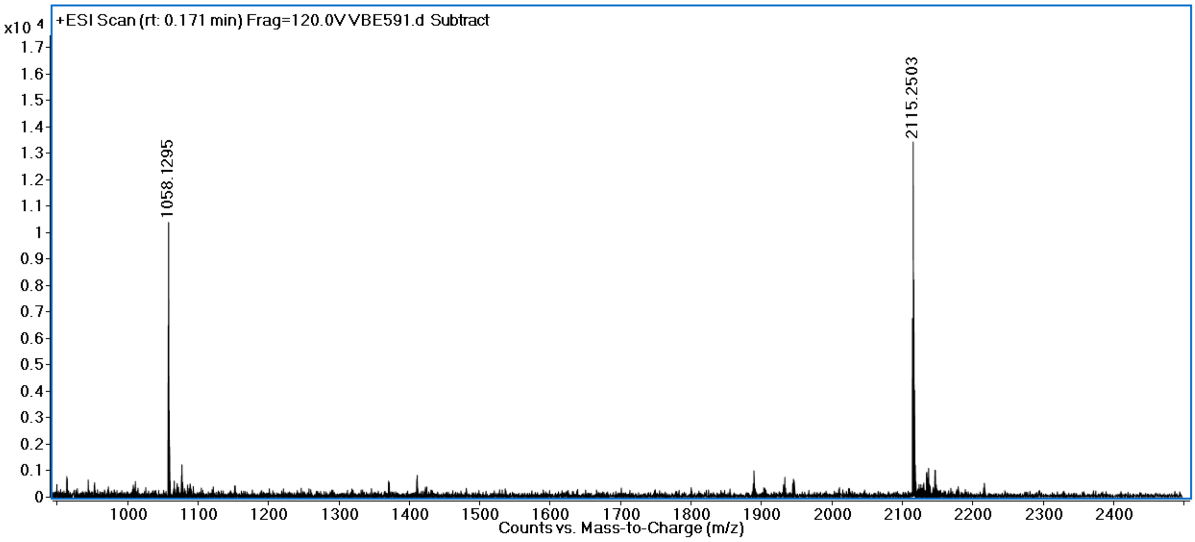

Figure S1. NMR ^{1}H (a), FTIR (b), ^{13}C (c) and ESI (d) spectra of *5,11,17,23-tetra(4-phtalimidomethyl-1,2,3-triazol-1-yl)-25,26,27,28-tetratetradecyloxycalix[4]arene* (**5**)

(a)

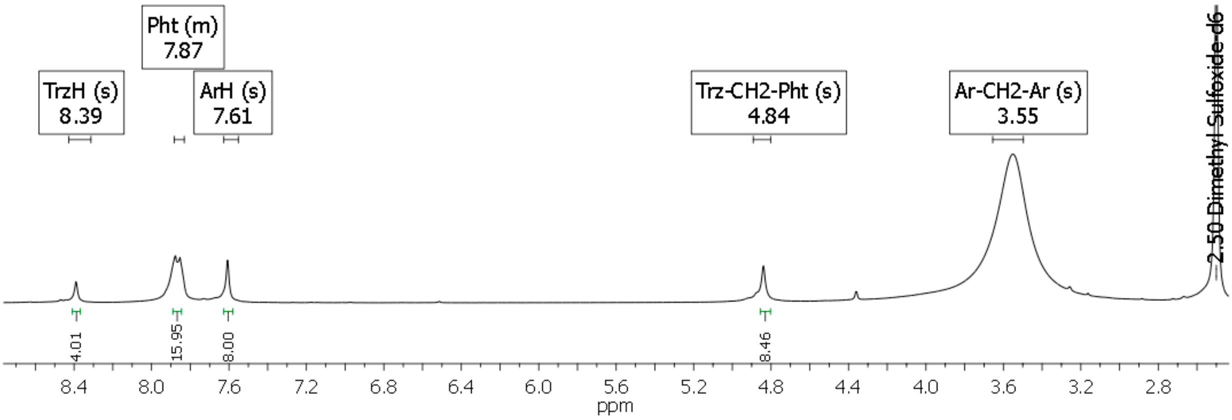

(b)

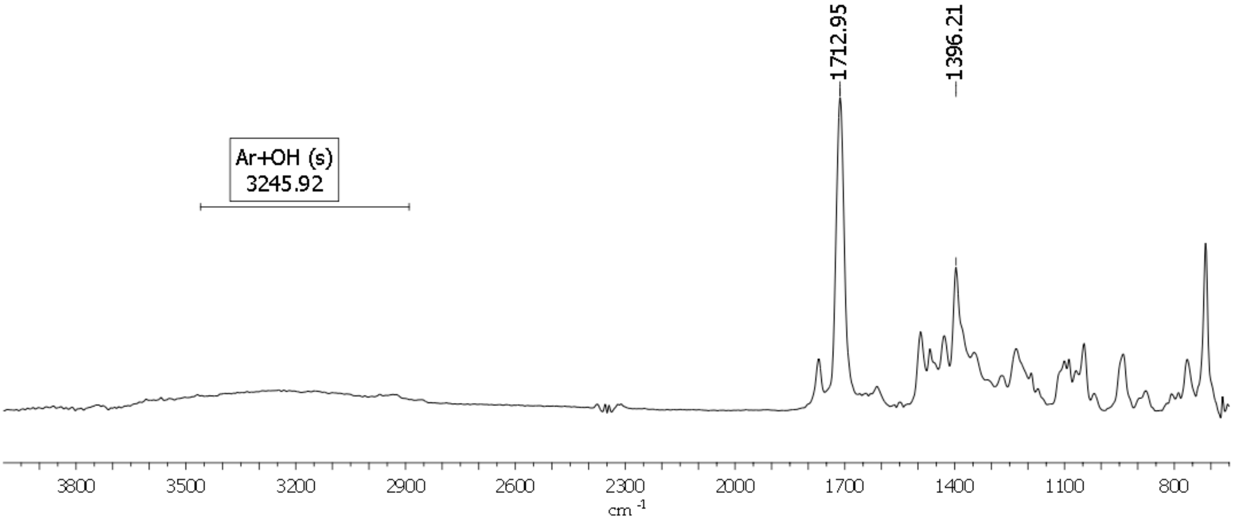

(c)

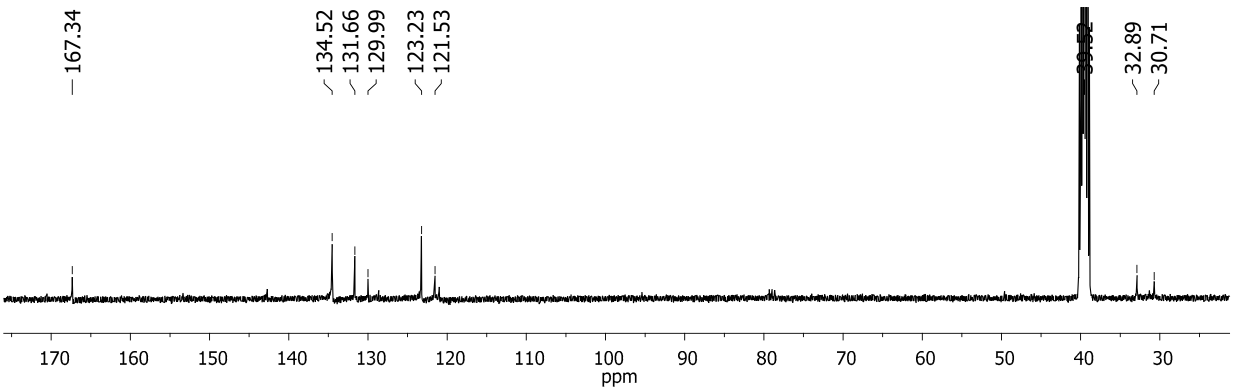

d)

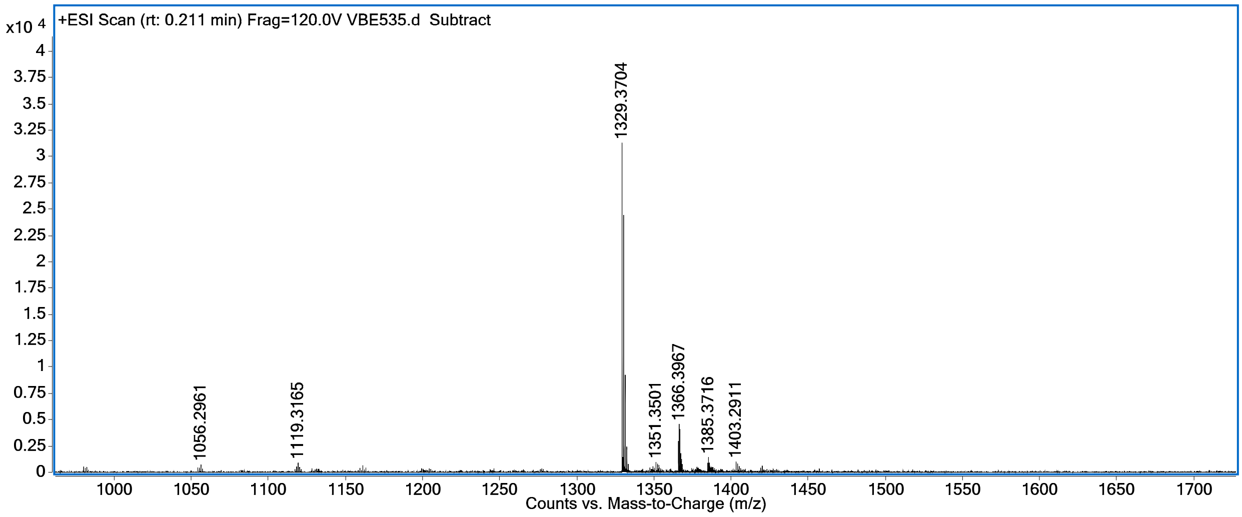

Figure S2. NMR ^{1}H (a), FTIR (b), ^{13}C (c) and HRESI MS (d) spectra of *5,11,17,23-tetra(4-phtalimidomethyl-1,2,3-triazol-1-yl)-25,26,27,28-tetrahydroxycalix[4]arene* (***6***).

a)

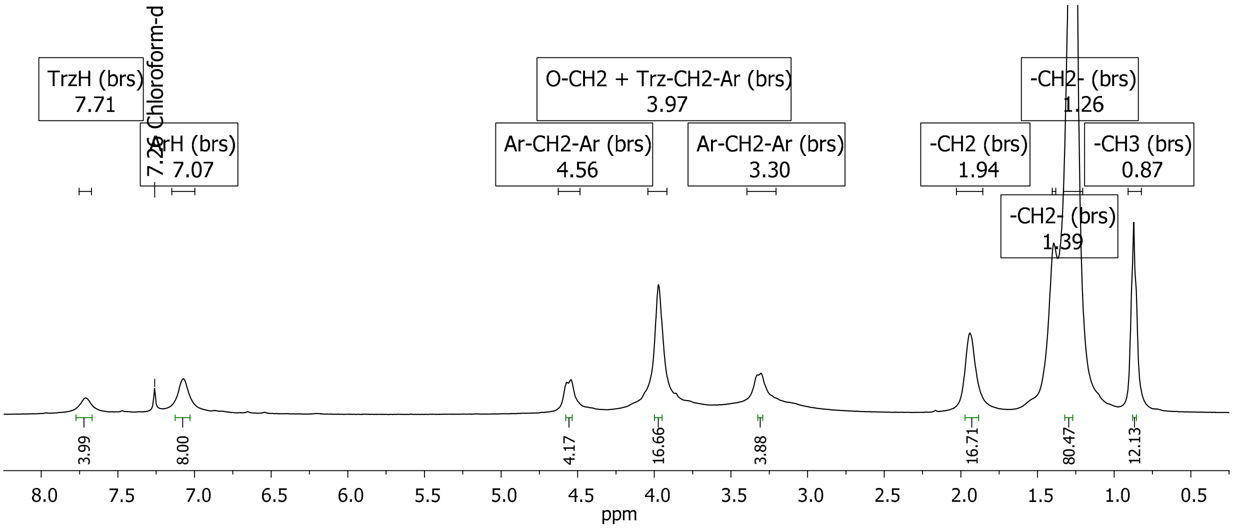

b)

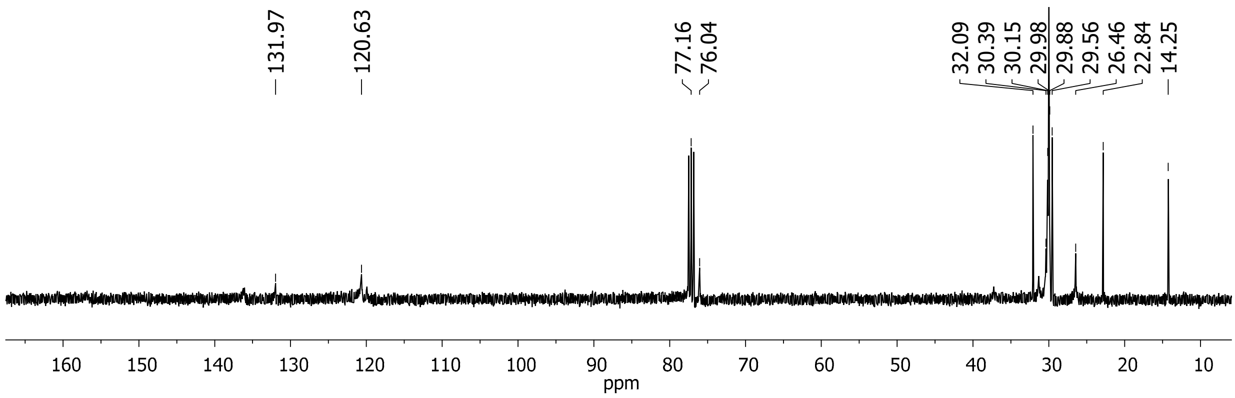

c)

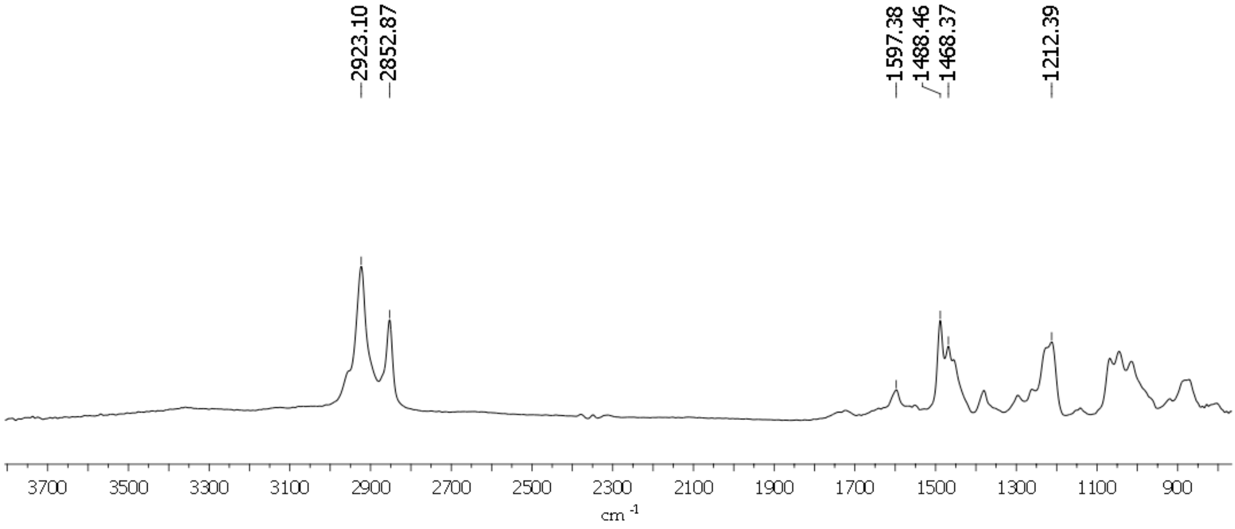

(d)

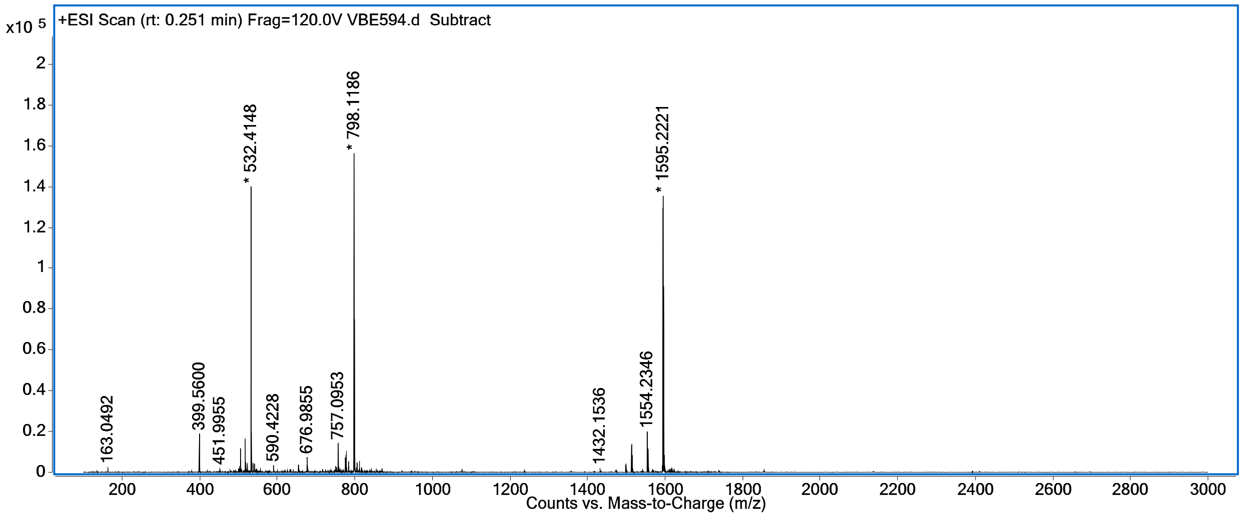

Figure S3. NMR ^{1}H (a), ^{13}C (b), FTIR (c) and ESI (d) spectra of *5,11,17,23-tetra(4-aminomethyl-1,2,3-triazol-1-yl)- 25,26,27,28-tetratetradecyloxycalix[4]arene tetrahydrochloride*

(a)

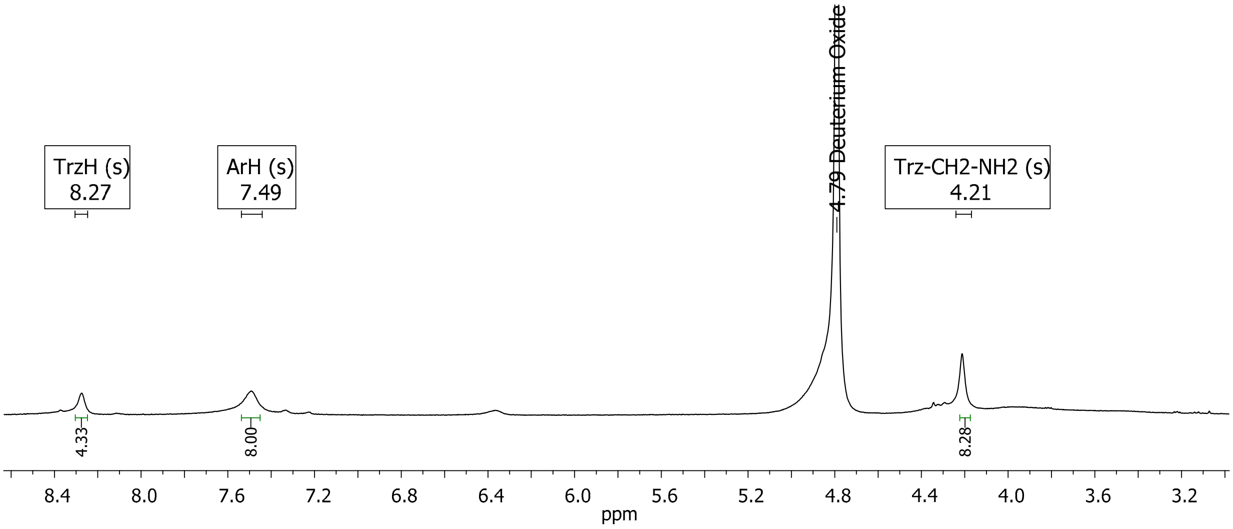

TrzH (s)
8.27
ArH (s)
7.49
4.79 Deuterium Oxide
Trz-CH2-NH2 (s)
4.21
4.33
8.00
8.28
8.4 8.0 7.6 7.2 6.8 6.4 6.0 5.6 5.2 4.8 4.4 4.0 3.6 3.2
ppm

(b)

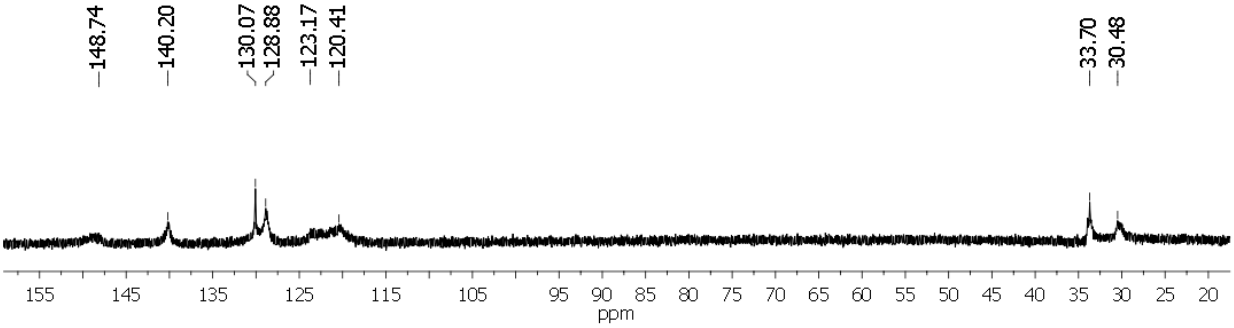

148.74
140.20
130.07
128.88
123.17
120.41
33.70
30.48
155 145 135 125 115 105 95 90 85 80 75 70 65 60 55 50 45 40 35 30 25 20
ppm

(c)

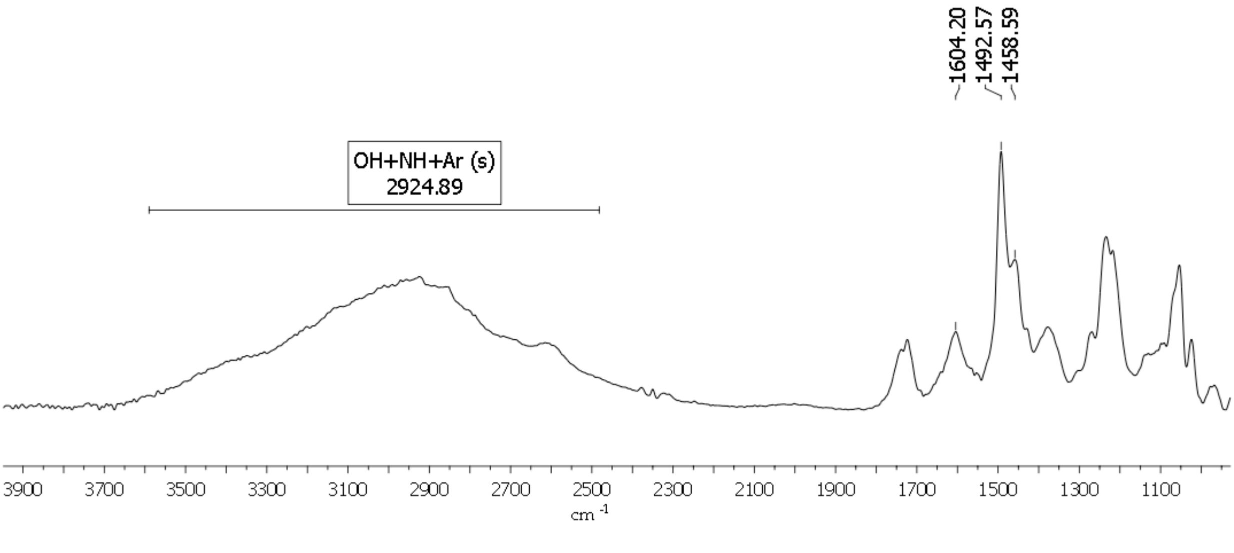

1604.20
1492.57
1458.59
OH+NH+Ar (s)
2924.89
3900 3700 3500 3300 3100 2900 2700 2500 2300 2100 1900 1700 1500 1300 1100
cm -1

(d)

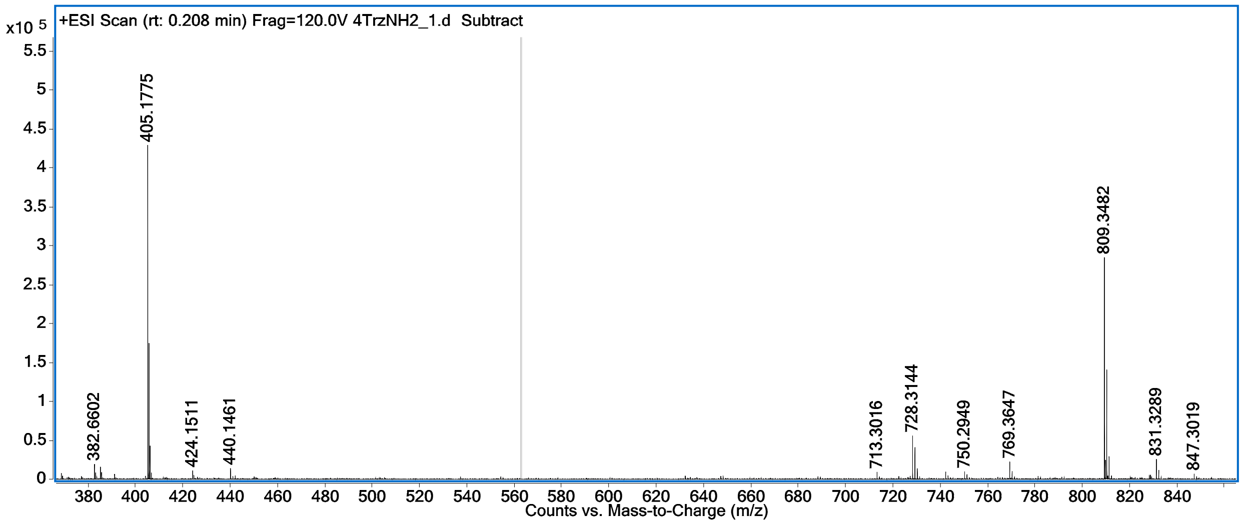

Figure S4. NMR ^{1}H (a), ^{13}C (b), FT IR (c) and HRESI MS (d) spectra of *5,11,17,23-tetra(4-aminomethyl-1,2,3-triazol-1-yl)- 25,26,27,28-tetrahydroxycalix[4]arene tetrahydrochloride.*

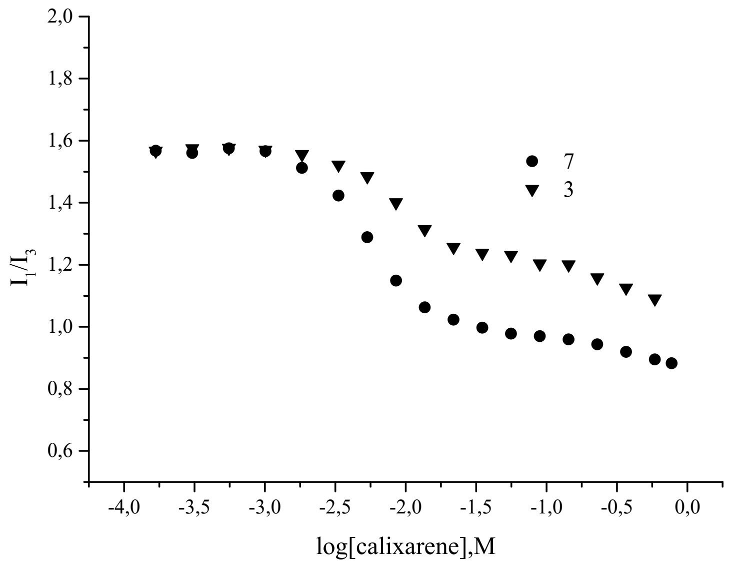

Figure S5. Dependence of the ratio of the first and third pyrene emission peaks on the logarithm of the concentration of macrocycles **3**, **7**.

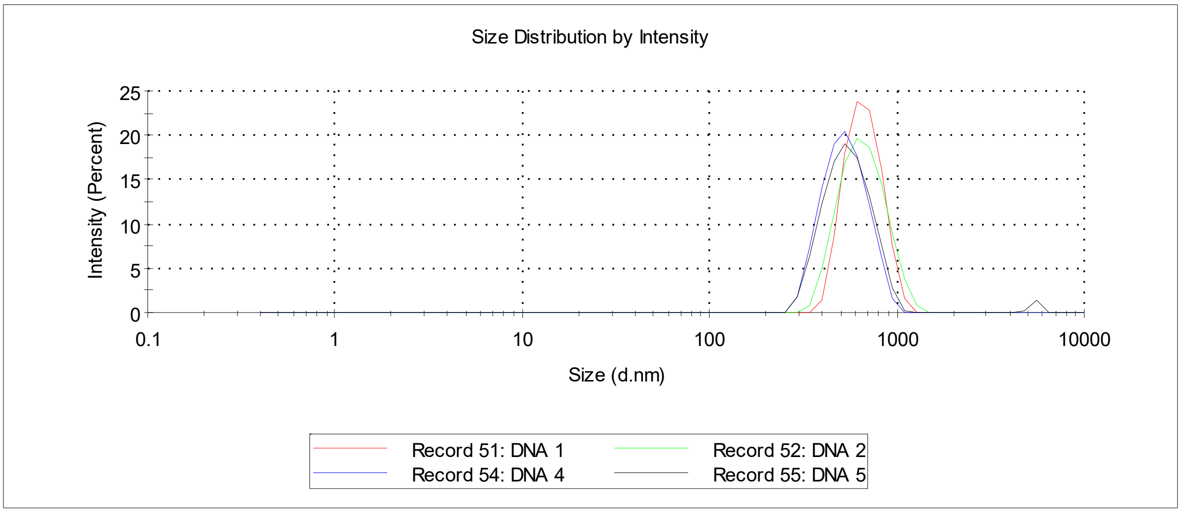
Size Distribution by Intensity
Intensity (Percent)
25
20
15
10
5
0
0.1
1
10
100
1000
10000
Size (d.nm)
Record 51: DNA 1
Record 52: DNA 2
Record 54: DNA 4
Record 55: DNA 5
(A)

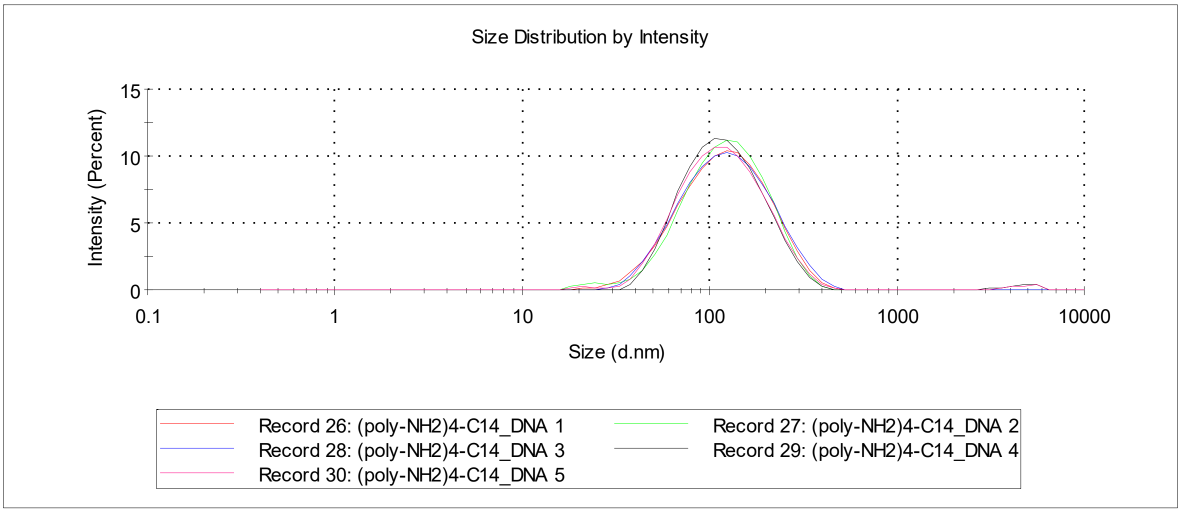
Size Distribution by Intensity
Intensity (Percent)
15
10
5
0
0.1
1
10
100
1000
10000
Size (d.nm)
Record 26: (poly-NH2)4-C14_DNA 1
Record 27: (poly-NH2)4-C14_DNA 2
Record 28: (poly-NH2)4-C14_DNA 3
Record 29: (poly-NH2)4-C14_DNA 4
Record 30: (poly-NH2)4-C14_DNA 5
(B)

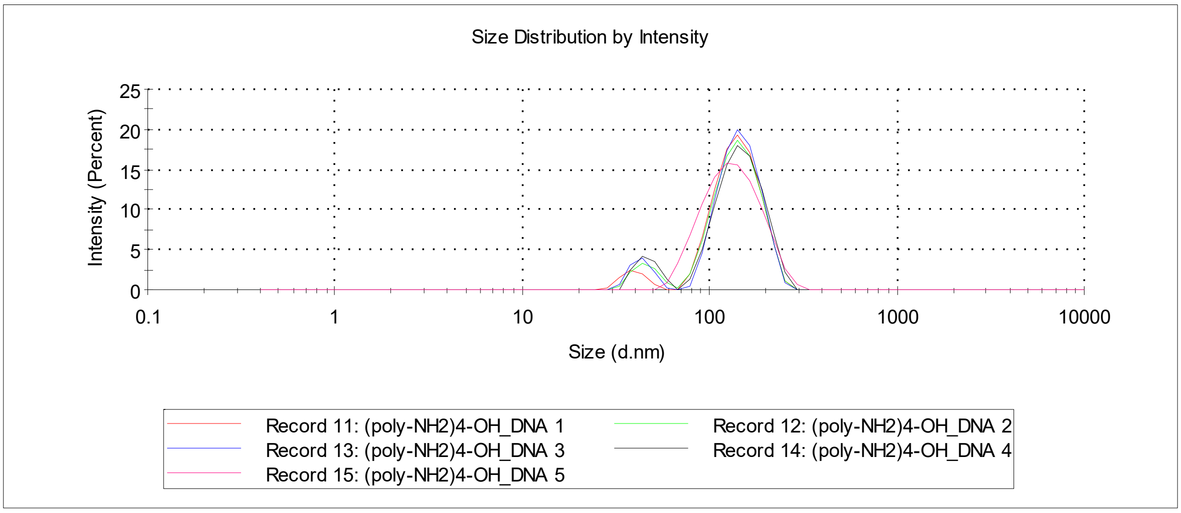
Size Distribution by Intensity
Intensity (Percent)
25
20
15
10
5
0
0.1
1
10
100
1000
10000
Size (d.nm)
Record 11: (poly-NH2)4-OH_DNA 1
Record 12: (poly-NH2)4-OH_DNA 2
Record 13: (poly-NH2)4-OH_DNA 3
Record 14: (poly-NH2)4-OH_DNA 4
Record 15: (poly-NH2)4-OH_DNA 5
(C)

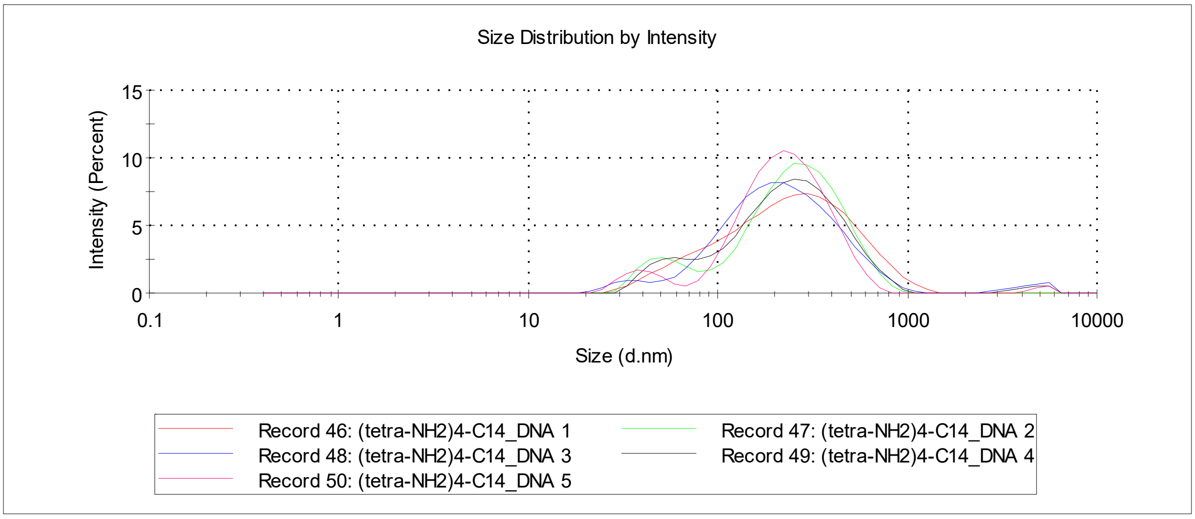

(D)

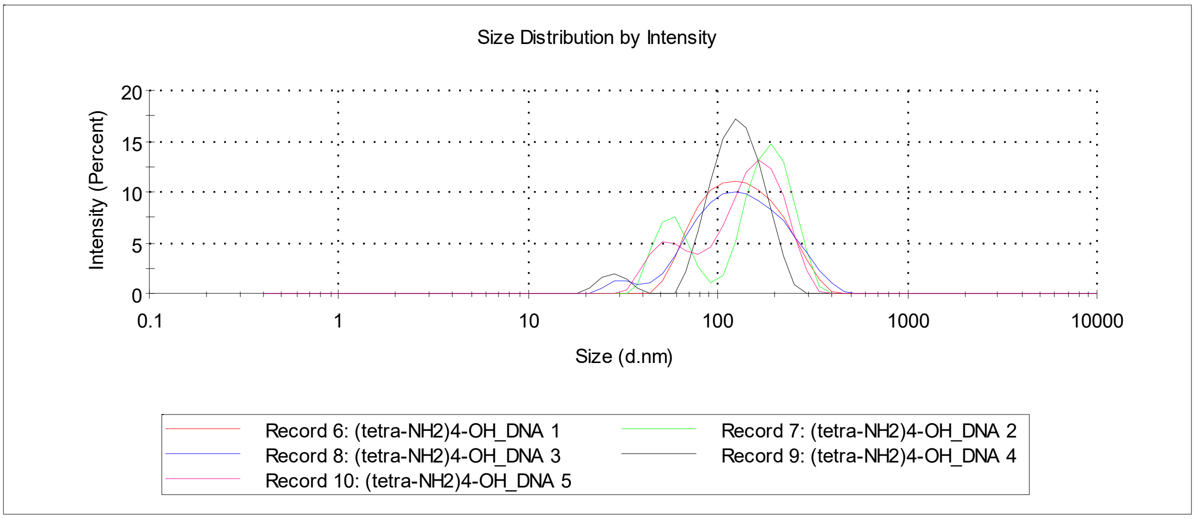

(E)

Figure S6. DLS intensity vs diameter graph for solutions of ct-DNA (A) and ct-DNA with **3** (B), **4**(C), **7**(D), **8** (E) ([ct-DNA]= 0.05 mM, [calixarene] = 0.1 mM,10 mM TRIS).

Cytotoxicity analysis of the compounds

This test is used to assess the metabolic activity of cells. 48 hours after the addition of the compounds, a medium was removed from each well and 100 µl of fresh medium with MTT reagent was added until the final MTT concentration of 0.5 mg/ml. Then the plates were placed in a CO_2 incubator at 37 °C for 4 hours. After that, 150 ml of dimethyl sulfoxide (DMSO) solution was added to the wells and incubated for another 15 minutes on a shaker. The optical density was measured at a wavelength of 590 nm using microplate reader Infinite M200 (Tecan, Switzerland). Cell viability was calculated as a relative value, taking the optical density values in the control wells as 100%. Data processing was carried out in the GraphPad Prism 8.
